# The collision of ChatGPT and traditional medicine: a perspective from bibliometric analysis

**DOI:** 10.1097/JS9.0000000000000662

**Published:** 2023-08-09

**Authors:** Zhimin Tan, Qiyu He, Shijian Feng

**Affiliations:** Department of Anaesthesiology and Urology, West China Hospital of Sichuan University, Chengdu, Sichuan Province, People’s Republic of China

*Dear Editor*,

Chat Generative Pre-trained Transformer (ChatGPT), an artificial intelligence (AI) language model, goes beyond its apparent capabilities of natural language processing and conversational interaction^1^. It is colliding with traditional clinical practice models, as researchers increasingly explore its integration into medicine. Timely summarization and reflection on current research are essential to effectively address future changes in this field.

A bibliometric study was conducted employing CiteSpace software to analyze published articles pertaining to ChatGPT. The primary objective of this study was to discern research hotspots and emerging trends, with the intention of furnishing healthcare professionals with pertinent references and guidance. We searched the Web of Science Core Collection with ‘ChatGPT’ as the keyword from its inception until June 2023. A total of 557 studies were included after removing duplicates and irrelevant articles, comprising 338 original articles, 37 review articles, and 192 correspondences and editorials. These studies originated from 65 countries or regions and formed interconnected author groups (Fig. 1A, B). Keyword co-occurrence analysis indicated a strong association with AI, reflecting the advent of the AI era (Fig. 1C). Clustering the included keywords using unsupervised machine learning highlighted research concentrations in the clinical practice, medical education, and medical research applications of ChatGPT (Fig. 1D).

**Figure 1:**
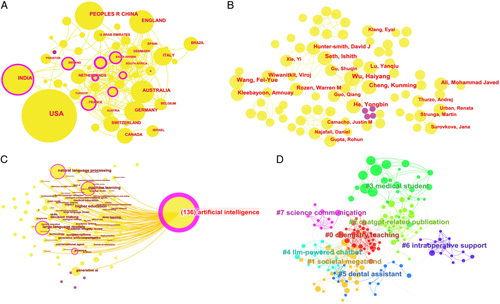
(A) Country co-occurrence network map; (B) author co-occurrence network map; (C) keyword co-occurrence network map; (D) clustering map of keywords.

The integration of ChatGPT into clinical practice is promising. Incorporating ChatGPT into monitoring systems provides surgeons with timely alerts, risk assessments, and predictive analytics, aiding early intervention and adverse event prevention^2^. ChatGPT can also analyze personalized patient data (genetic information, biomarkers, treatment history) to generate tailored treatment recommendations and predict individual responses. Seamless integration into existing clinical workflows and electronic health record (EHR) systems is essential for ChatGPT’s effective use. This enables efficient real-time clinical decision support and documentation. Future research should explore integrating ChatGPT into EHR systems for intelligent data extraction, summarization, and analysis, supporting clinical research, healthcare quality improvement, and evidence-based medicine.

The possibility of ChatGPT in medical education is being explored^3^. It can assist medical students as a self-learning tool, simulate clinical scenarios, and contribute as a virtual team member. Physicians can consult ChatGPT for up-to-date medical research, treatment guidelines, and clinical practices, advancing their continuing education. Additionally, ChatGPT can educate patients by addressing inquiries, providing health education, aiding in treatment decisions, facilitating patient triage, conducting preliminary assessments, offering remote guidance for home care, and ultimately empowering patients to understand their conditions and actively participate in their treatment.

ChatGPT enhances the efficiency of medical research by facilitating literature reviews and knowledge organization. It assists researchers in staying updated on the latest advancements and research trends in specific fields. Additionally, ChatGPT enables the analysis of extensive medical data, such as clinical trial results, case reports, and medical imaging, for data interpretation, pattern recognition, and correlation analysis. This drives progress in medical research and ultimately improves patient outcomes.

The clinical application of ChatGPT poses potential risks and challenges, considering the inclusion of sensitive patient data. Therefore, its use in medical decision-making must comply with ethical guidelines and legal regulations. Healthcare professionals and researchers should define ChatGPT’s role and limitations in medical practice, ensuring patient informed consent and confidentiality^4^. Ethical review boards should strengthen their evaluation and supervision of ChatGPT research and applications. Moreover, enhancing the transparency and interpretability of ChatGPT’s algorithms is necessary, given its complex deep learning model.

In conclusion, the collision between ChatGPT and traditional medicine is highly significant, with the potential to improve healthcare quality, patient outcomes, and advance medical education and research. However, it also poses challenges and risks that necessitate careful consideration in its utilization.

## Ethical approval

Not applicable.

## Sources of funding

None.

## Author contribution

Z.T.: conceptualization, data acquisition, and writing; Q.H.: statistical analysis, creation of figures, and writing; S.F.: review, editing, and supervision.

## Conflicts of interest disclosure

The authors declare that they no conflicts of interest.

## Research registration unique identifying number (UIN)

None.

## Guarantor

All authors.

## Data availability statement

Data are available from the corresponding author if the justification for the requirement is justified.
